# Synthesis and Antiviral Activity of *N*-Phenylbenzamide Derivatives, a Novel Class of Enterovirus 71 Inhibitors

**DOI:** 10.3390/molecules18033630

**Published:** 2013-03-21

**Authors:** Xing-Yue Ji, Hui-Qiang Wang, Lan-Hu Hao, Wei-Ying He, Rong-Mei Gao, Yan-Ping Li, Yu-Huan Li, Jian-Dong Jiang, Zhuo-Rong Li

**Affiliations:** Institute of Medicinal Biotechnology, Chinese Academy of Medical Science and Peking Union Medical College, Beijing 100050, China

**Keywords:** synthesis, EV 71, *N*-phenylbenzamide

## Abstract

A series of novel *N*-phenylbenzamide derivatives were synthesized and their anti-EV 71 activities were assayed *in vitro*. Among the compounds tested, 3-amino-*N*-(4-bromophenyl)-4-methoxybenzamide (**1e**) was active against the EV 71 strains tested at low micromolar concentrations, with IC_50_ values ranging from 5.7 ± 0.8–12 ± 1.2 μM, and its cytotoxicity to Vero cells (TC_50_ = 620 ± 0.0 μM) was far lower than that of pirodavir (TC_50_ = 31 ± 2.2 μM). Based on these results, compound **1e** is a promising lead compound for the development of anti-EV 71 drugs.

## 1. Introduction

Enterovirus 71 (EV 71) is a single-strained and positive sense RNA virus that belongs to the *Picornaviridae* family. It was first isolated and identified from patients with central nervous system (CNS) diseases in California between 1969 and 1974 [1]. Since then, outbreaks of EV 71 have been reported in several countries, especially in the Asia Pacific region [2,3,4]. Diseases caused by an EV 71 infection include hand-foot-mouth disease (HFMD), herpangina (HA) and CNS involvement with fatal pulmonary edema [5]. EV 71 infections are a global public health problem causing severe clinical illness and even death in young children. However, there are currently no antiviral drugs or vaccines available in the clinic against EV 71, and the majority of treatments are simply supportive symptom management treatments. Consequently, there is an increasing need for the discovery of effective drugs against EV 71. Additionally, due to the high mutation rate of EV 71, several genotypes have been isolated in the clinic [6], and, at times, more than one genotype has been associated with both complicated and uncomplicated diseases [7]. Therefore, pinpointing the active strain has been unsuccessful. Consequently, the development of anti-EV 71 drugs should be mainly focused on the discovery of compounds with activity against several genotypes of EV 71.

**Figure 1:**
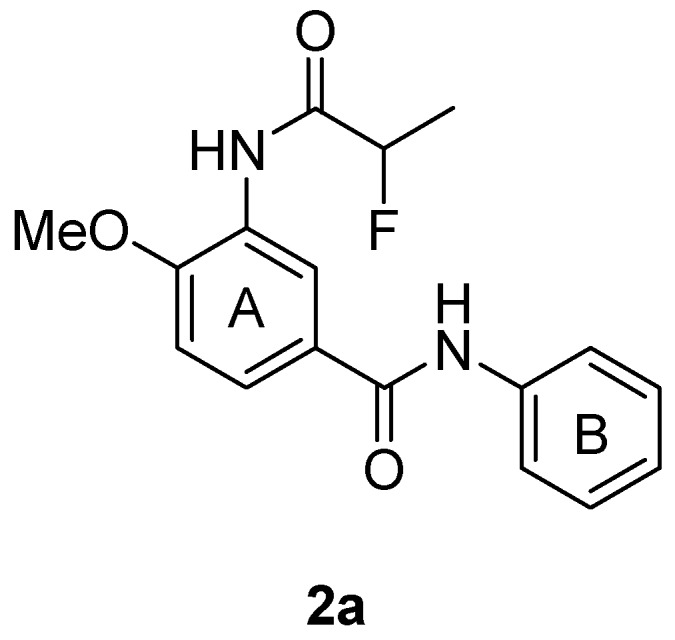
The structure of compound **2a**.

Antiviral screening of our privileged structure library was carried out, and compound **2a** (Figure 1) exhibited moderate activity against EV 71 virus (strain H, C2 genotype) *in vitro* with an IC_50_ value of 18 ± 1.2 μM. In order to obtain compounds with more potent activity and superior physicochemical profiles, we synthesized a series of *N*-phenylbenzamide derivatives and evaluated their antiviral activity against four strains (SZ-98, JS-52-3, H, BrCr) of EV 71 in Vero cells. The structure modification was mainly made to the substituents at the C-3 position of the benzene ring A to investigate the importance of amide group for the anti-EV 71 activity. Meanwhile, because there was no substituent on the ring B of compound **2a**, which possibly made it vulnerable to metabolism, we also synthesized derivatives with substituents on the ring B to make them metabolically stable, and also to explore the structure-activity relationships on the ring B. This is the first report of the anti-EV 71 activity of the *N*-phenylbenzamide scaffold, to the best of our knowledge.

## 2. Results and Discussion

### 2.1. Chemistry

**Scheme 1:**
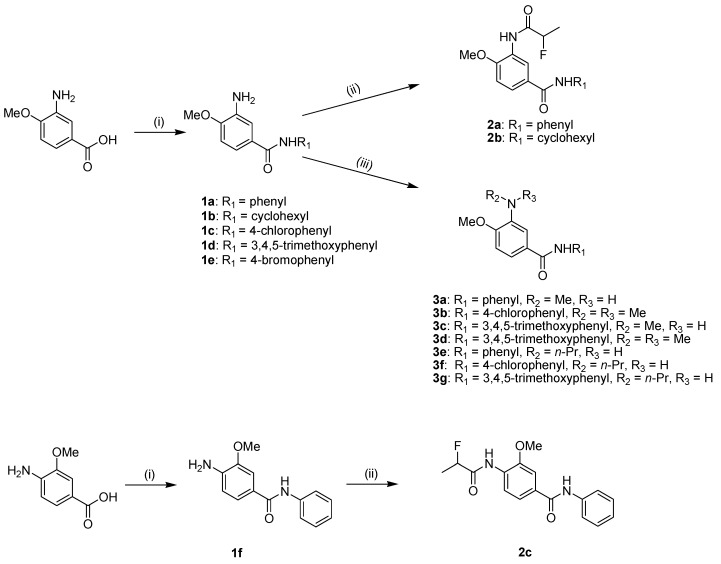
The synthetic route to compounds **2a**–**c** and **3a**–**g**.

**Scheme 2:**
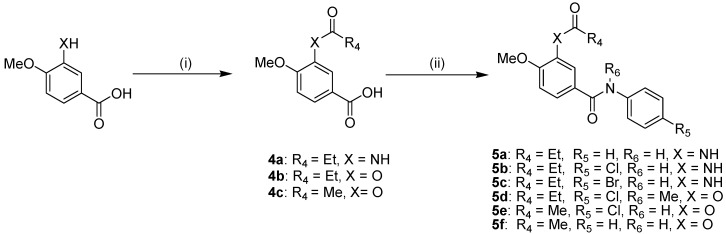
The synthetic route to compounds **5a**–**f**.

As shown in Scheme 1 and Scheme 2, a series of *N*-phenylbenzamide derivatives were designed and synthesized according to our previous reported method [8]. In Scheme 1, 3-amino-4-methoxybenzoic acid was chosen as the starting material and was condensed with a variety of amines, using *N,N′*-diisopropylcarbodiimide (DIC) as the coupling reagent and *N*-hydroxybenzotriazole (HOBt) as the activating reagent [9], to yield the intermediate compounds **1a**–**e**. Under the same conditions, compounds **1a**, **b** were condensed with 2-fluoropropionic acid, which was obtained by the hydrolysis of methyl 2-fluoropropanoate, to afford the target compounds **2a**, **b**. Alkylation of the amine groups of compounds **1a**, **c** and **d** was accomplished by a nucleophilic substitution reaction. To avoid cleavage of the amide bond during the reaction, a mild base (NaHCO_3_) was employed as the acid-binding agent. When iodomethane (MeI) was used as the alkylating agent in excess, dialkylation products **3b**, **d** were also obtained in addition to monoalkylated products. Additionally, by a similar method used to synthesize compounds **2a**, **b**, we also synthesized compound **2c** using 3-methoxy-4-aminobenzoic acid as the starting material.

*Reagents and conditions*: (i) DIC, HOBt, R_1_NH_2_, CH_2_Cl_2_, rt, 5 h, 60–70%, (ii) 2-fluoropropionic acid, DIC, HOBt, CH_2_Cl_2_, rt, 5 h, 60–70%, (iii) MeI/PrI, NaHCO_3_, CH_2_Cl_2_, rt, 12 h, 30–50%.

*Reagents and conditions*: (i) TEA, CH_2_Cl_2_, R_4_COCl, 0 °C-rt, 6 h, 70–80%, (ii) DIC, HOBt, amines, CH_2_Cl_2_, rt, 5 h, 60–70%, or SOCl_2_, reflux 1 h, then CH_2_Cl_2_, TEA, amines , 0 °C-rt, 6 h, 60–70%.

As depicted in Scheme 2, we also synthesized several derivatives with propionyloxy (acetoxy) and propionylamino substituents on the benzene ring A. 3-Hydroxyl/amino-4-methoxybenzoic acid was first reacted in dichloromethane with propionyl chloride or acetyl chloride using triethylamine (TEA) as the base to afford compounds **4a**–**c**. By using a similar method to that used for compounds **1a**–**e**, compounds **5a**–**f** were thus obtained. Alternatively, compounds **4a**–**c** were first reacted with thionyl chloride under refluxing conditions to give intermediate chloride products, which were condensed with a substituted arylamine to yield the target compounds **5a**–**f**. Ultimately, compounds **5a**–**f** were obtained in overall yields of 40–60%.

### 2.2. Anti-EV 71 Activity

**Figure 2:**
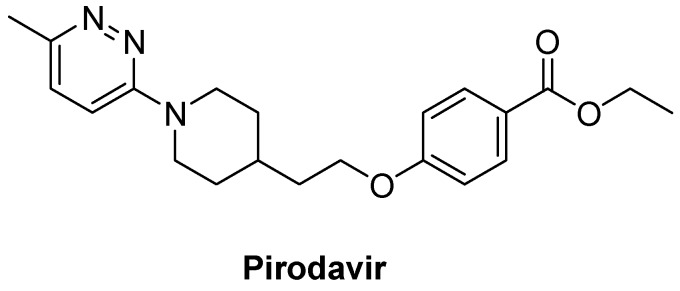
The chemical structure of pirodavir.

The EV 71 was divided into three genotypes A, B and C based on nucleotide sequence comparisons, and genotypes B and C were subdivided into B1-B5 and C1-C5 [6,10]. Genotype C is the most common genotype found in mainland China, especially the C4 subgenotype [4,11]. Consequently, the intermediate compounds **1a**, **1c** and **1e**, and the final products **2a**–**c**, **3a**–**g**, and **5a**–**f** were evaluated for their anti-EV 71 activity in Vero cells against genotypes C4 (strains SZ-98 and JS-52-3), C2 (strain H) and A (strain BrCr). Pirodavir (Figure 2) is a rather potent and broad-spectrum picornavirus inhibitor. It was reported to be effective against 80% of 16 enteroviruses tested at the concentration of 1.3 μg/mL [12]. Therefore, we employed pirodavir as positive control to validate our model. The IC_50_ values were calculated by the Reed and Muench method, and the cytotoxicity was monitored by the CPE method.

As indicated in Table 1, the cytotoxicity of all the synthesized compounds was far lower than that of pirodavir, and most of the synthesized *N*-phenylamide derivatives exhibited some activity against several strains of EV 71 simultaneously, especially compound **1e**, which was active against all the strains tested at low micromolar concentrations (5.7 ± 0.80–12 ± 1.2 μM). In addition to compound **1e**, other compounds also showed moderate levels of inhibitory activity with IC_50_ values of approximately 15 μM including, compound **1c** against strain SZ-98, compound **3g** against strains H and BrCr, **3e** against strain H, **5c** against strains SZ-98, JS-52-3 and H, and compound **5e** against strain SZ-98. Moreover, the selectivity index (SI) values of compounds **1c** (10–36), **1e** (51–110), **3g** (16–35), **5b** (>16 – >27), **5c** (16–63) and **5e** (15–37) were also comparable or superior to those of pirodavir (25–52).

**Table 1:** The activity of the synthesized *N*-phenylbenzamide derivatives against several strains of EV 71.

Cpds	TC_50_ (μM)	SZ-98		JS-52-3		H		BrCr	
		IC_50_ (μM)	SI	IC_50_ (μM)	SI	IC_50_ (μM)	SI	IC_50_ (μM)	SI
**1a**	>820	350 ± 24	2.3	160 ± 7.9	5.2	190 ± 14	4.3	160 ± 12	5.2
**1c**	520 ± 29	15 ± 0.6	36	46 ± 5.7	12	34 ± 3.6	16	56 ± 4.2	10
**1e**	620 ± 0.0	12 ± 1.1	51	9.8 ± 0.4	64	5.7 ± 0.8	110	9.1 ± 1.4	68
**2a**	630 ± 0.0	>630	-	160 ± 5.7	>3.9	18 ± 1.2	>35	>630	-
**2b**	>620	>620	-	210 ± 0.0	>3.0	430 ± 36	>1.4	360 ± 26	>1.7
**2c**	>630	110 ± 12	>5.8	34 ± 2.8		41 ± 2.9	>16	90 ± 9.5	>7.0
**3a**	780 ± 0.0	>260	-	260 ± 0.0	3.0	260 ± 0.0	3.0	180 ± 14	4.3
**3b**	510 ± 38	73 ± 8.6	7.0	42 ± 3.1	12	31 ± 4.3	16	50 ± 3.8	10
**3c**	280 ± 5.9	64 ± 0.0	4.3	37 ± 1.9	7.5	37 ± 1.2	7.5	44 ± 1.0	6.2
**3d**	>560	320 ± 32	>1.7	>560	-	190 ± 0.0	>3.0	190 ± 0.0	>3.0
**3e**	410 ± 22	>78	-	34 ± 4.6	12	20 ± 1.2	20	45 ± 0.0	9.0
**3f**	120	>70	-	>70	-	>70	-	>70	-
**3g**	530	>59	-	34 ± 2.5	16	15 ± 0.0	35	20 ± 3.1	27
**5a**	>670	>75	-	58 ± 4.9	>12	96 ± 10	>7.0	45 ± 10	9.0
**5b**	>600	38 ± 2.9	> 16	39 ± 1.9	>16	22 ± 2.9	>27	>67	-
**5c**	530 ± 0.0	11 ± 0.8	47	20 ± 3.1	27	8.4 ± 1.2	63	34 ± 0.0	16
**5d**	280 ± 21	190 ± 12	1.4	44 ± 3.7	6.2	65 ± 0.0	4.3	53 ± 2.4	5.2
**5e**	470 ± 22	13 ± 2.4	37	32 ± 3.6	14	22 ± 4.7	21	32 ± 3.9	15
**5f**	360 ± 39	90 ± 11	4.0	98 ± 4.7	3.7	47 ± 6.1	7.6	95 ± 9.0	3.8
Pirodavir	31 ± 2.2	1.2 ± 0.2	25	1.0 ± 0.2	30	0.6 ± 0.1	52	1.0 ± 0.2	30

TC_50_ was defined as the concentration that inhibits 50% cellular growth in comparison with untreated controls and calculated by Reed and Muench method. IC_50_ (50% inhibitory concentration) was calculated by Reed & Muench method. The selectivity index (SI) was calculated as the ratio of TC_50_/IC_50_. “-” stands for no antiviral selectivity.

In terms of structure-activity relationships, benzene ring B was essential for anti-EV 71 activity. For example, when benzene ring B was replaced by a cyclohexyl group (**2b**), no anti-EV 71 activity was observed. On the basis of the activity of compounds **1c**, **1e**, **5b**, **5c** and **5e** against EV 71, it was concluded that the introduction of electron-withdrawing groups (Cl and Br, especially Br) at the *para* position of benzene ring B increased anti-EV 71 activity. Substitution of acetoxy groups (**5e**, **f**) for the propionylamino group (**5a**, **b**) on benzene ring A had little influence on anti-EV 71 activity. Because the activity of compounds with propionyl substituent at the amino group (**5b**, **c**) was slightly inferior to their unsubstituted counterparts (**1c**, **e**), it can be concluded that the propionyl substituent at the amino group disfavors the anti-EV 71 activity. Compound **5d** presented less potent activity against EV 71 in comparison to **5e**. Therefore, it is possible that H-bond donor group at the position of R_6_ substituent favors the anti-EV 71 activity.

## 3. Experimental

### 3.1. General

All reagents and solvents were purchased from J&K and Alfa Aesar Chemicals, and were used without purification. ^1^H-NMR and ^13^C-NMR spectra were recorded in CDCl_3_ or DMSO-*d*_6_ on a Varian Inova 400/500 MHz spectrometer (Varian, San Francisco, CA, USA). Chemical shift was reported in parts per million relative to tetramethylsilane as the internal standard. Melting points were determined with a X6 microscope melting point apparatus and were uncorrected. Electrospray ionisation (ESI) high-resolution mass spectra (HRMS) were obtained on an MDS SCIEX Q-Trap mass spectrometer. The area normalization purities of the tested compounds were >95% as determined using analytical high-performance liquid chromatography (HPLC method for all compounds: detector wavelength: 260 nm, column temperature: 25 °C, column: ODS (5 μm, 4.6 × 250 mm), mobile phase: methanol/water = 75/25, flow rate = 1 mL/min). For the synthesis and spectral data of compounds **1a**, **2a**, **4a**, **4b**, **5a** and **5c**, please refer to our previous paper [8].

#### 3.1.1. General Procedure for the Synthesis of Compound **1b**–**f**, **5b**, **5e**, **5f**

3-Amino-4-methoxybenzoic acid/**4a**, **c** (1.20 mmol) was dissolved in CH_2_Cl_2_ (20 mL), and DIC (1.82 mmol) and HOBt (1.82 mmol) was added to the solution. The resulting mixture was stirred for 0.5 h at room temperature, after which amines (1.68 mmol) was added. After approximately 12 h, the reaction was quenched by the addition of 0.5 N NaOH solution (20 mL), and the organic layer was separated and washed with dilute hydrochloric acid (10%, 30 mL) and brine (30 mL), successively. The solution was then dried over anhydrous MgSO_4_, filtered and concentrated, and the crude residue was purified over silica gel (petroleum/ethyl acetate = 3/2) to give the corresponding compounds.

*3-Amino-N-cyclohexyl-4-methoxybenzamide* (**1b**). White solid, yield: 65%. mp: 171–172 °C. ^1^H-NMR (400 MHz, DMSO-*d_6_*) δ: 1.13 (1H, m), 1.24 (4H, m), 1.62 (1H, m), 1.75 (4H, m), 3.63 (1H, m), 3.79 (3H, s), 4.79 (2H, br), 6.80 (1H, d, *J* = 8.4 Hz), 7.07 (1H, d, *J* = 8.4 Hz), 7.12 (1H, s), 8.25 (1H, s). ESI-HRMS *m/z*: 249.16018 (M+H^+^) (Calcd for C_1__4_H_21_N_2_O_2_: 249.16030). 

*3-Amino-N-(4-chlorophenyl)-4-methoxybenzamide* (**1c**). White solid, yield: 66%. mp 152–154 °C. ^1^H-NMR (400 MHz, DMSO-*d_6_*) δ: 3.84 (s, 3H), 5.46 (2H, br), 6.88 (d, 1H, *J* = 6.8 Hz), 7.19 (m, 2H), 7.37 (d, 2H, *J* = 8.0Hz), 7.78 (d, 2H, *J* = 8.0Hz), 10.07 (s, 1H). ^13^C-NMR (100 MHz, CDCl_3_) δ: 55.6, 109.6, 113.4, 117.4, 121.2, 124.7, 128.9, 129.2, 136.5, 150.1, 156.9. ESI-HRMS *m/z*: 277.07487 (M+H^+^) (Calcd for C_1__4_H_14_ClN_2_O_2_: 277.07438).

*3-Amino-4-methoxy-N-(3,4,5-trimethoxyphenyl)benzamide* (**1d**). White solid, yield: 65%. mp: 140–142 °C. ^1^H-NMR (500 MHz, DMSO-*d_6_*) δ: 3.62 (3H, s), 3.76 (6H, s), 3.83 (3H, s), 4.92 (2H, s), 6.88 (1H, d, *J* = 8.0 Hz), 7.22 (4H, m), 9.85 (1H, s). ESI-HRMS *m/z*: 333.14778 (M+H^+^) (Calcd for C_17_H_21_N_2_O_5_: 333.14505).

*3-Amino-N-(4-bromophenyl)-4-methoxybenzamide* (**1e**). White solid, yield: 68%. mp 190–192°C. ^1^H-NMR (500 MHz, DMSO-*d_6_*) δ: 3.84 (s, 3H), 4.97 (s, 2H), 6.90 (d, 1H, *J* = 8.0 Hz), 7.21 (m, 2H), 7.51 (d, 2H, *J* = 8.0 Hz), 7.74 (d, 2H, *J* = 8.0 Hz), 10.10 (s, 1H). ^13^C-NMR (100 MHz, CDCl_3_) δ: 55.6, 109.6, 113.6, 116.6, 117.3, 121.5, 127.3, 131.9, 136.5, 137.3, 150.1, 165.5. ESI-HRMS *m/z*: 321.02365 (M+H^+^) (Calcd for C_14_H_14_BrN_2_O_2_: 321.02387).

*4-Amino-3-methoxy-N-phenylbenzamide* (**1f**). White solid, yield: 70%. mp: 174–176°C. ^1^H-NMR (400 MHz, DMSO-*d_6_*) δ: 3.82 (s, 3H), 5.37 (2H, s), 6.66 (1H, d, *J* = 8.0 Hz), 7.03 (1H, d, *J* = 8.0 Hz), 7.32 (2H, m), 7.45 (2H, m), 7.73 (2H, d, *J* = 7.6 Hz), 9.77 (1H, s). ESI-HRMS *m/z*: 243.11329 (M+H^+^) (Calcd for C_1__4_H_15_N_2_O_2_: 243.11335).

*N-(4-Chlorophenyl)-4-methoxy-3-propionamidobenzamide* (**5b**). White solid, yield: 70%. mp: 130–132 °C. ^1^H-NMR (400 MHz, CDCl_3_) δ: 1.07 (3H, t, *J* = 7.6 Hz), 2.42 (2H, q, *J* = 7.6 Hz), 3.96 (3H, s), 6.98 (1H, d, *J* = 8.0 Hz), 7.32 (1H, d, *J* = 8.0 Hz), 7.62 (2H, d, *J* = 8.0 Hz), 7.79 (1H, dd, *J* = 8.0 and 2.0 Hz), 7.82 (1H, s), 8.09 (1H, s), 8.87 (1H, s). ESI-HRMS m/z: 333.10200 (M+H^+^) (Calcd for C_17_H_18_ClN_2_O_3_: 333.10059).

*N-(4-Chlorophenyl)-4-methoxy-3-acetoxybenzamide* (**5e**). White solid, yield: 58%. mp: 177–179 °C. ^1^H-NMR (400 MHz, DMSO-*d_6_*) δ: 2.34 (3H, s), 3.89 (3H, s), 6.73 (1H, d, *J* = 8.0 Hz), 7.01 (2H, d, *J* = 8.4 Hz), 7.13 (1H, d, *J* = 8.0 Hz), 7.22 (2H, d, *J* = 8.4 Hz), 8.30 (1H, s), 9.01 (1H, s). ESI-HRMS m/z: 320.06891 (M+H^+^) (Calcd for C_1__6_H_1__5_ClNO_4_: 320.06896).

*4-Methoxy-N-phenyl-3-acetoxybenzamide* (**5f**). White solid, yield: 61%. mp: 153–155 °C. ^1^H-NMR (400 MHz, DMSO-*d_6_*) δ: 2.29 (3H, s), 3.85 (3H, s), 7.08 (1H, t, *J* = 8.0 Hz), 7.26 (1H, d, *J* = 8.4 Hz), 7.33 (2H, t, *J* = 8.0 Hz), 7.73 (3H, m), 7.92 (d, 1H, *J* = 8.4 Hz), 10.11 (1H, s). ESI-HRMS m/z: 286.10799 (M+H^+^) (Calcd for C_1__6_H_1__6_NO_4_: 286.10793).

#### 3.1.2. General Procedure for the Synthesis of Compounds **2b**, **c**

2-Fluoropropanoic acid (2.40 mmol) was dissolved in CH_2_Cl_2_ (20 mL), and DIC (3.60 mmol) and HOBt (3.60 mmol) was added to the solution. The resulting mixture was stirred for 0.5 h at room temperature, after which compound **1b/1e** (1.71 mmol) was added. After approximately 12 h, the reaction was quenched by the addition of 0.5 N NaOH solution (20 mL), and the organic layer was separated and washed with dilute hydrochloric acid (10%, 30 mL) and brine (30 mL), successively. The solution was then dried over anhydrous MgSO_4_, filtered and concentrated, and the crude residue was purified over silica gel (petroleum/ethyl acetate = 4/1) to give compounds **2b**, **c**.

*N-Cyclohexyl-3-(2-fluoropropanamido)-4-methoxybenzamide* (**2b**). White solid, yield: 73%. mp: 192–194 °C. ^1^H-NMR (400 MHz, DMSO-*d_6_*) δ: 1.15 (1H, m), 1.26 (4H, m), 1.55 (3H, dd, *J* = 6.4 and 16.8 Hz), 1.61 (1H, m), 1.72 (4H, m), 3.73 (1H, m), 3.93 (3H, s), 5.34 (1H, qq, *J* = 6.4 and 48.8 Hz), 7.15 (1H, d, *J* = 8.0 Hz), 8.06 (1H, d, *J* = 8.0 Hz),8.33 (1H, s), 9.16 (1H, s), 13.63 (1H, s). ESI-HRMS *m/z*: 323.17695 (M+H^+^) (Calcd for C_17_H_24_FN_2_O_3_: 323.17710).

*4-(2-Fluoropropanamido)-3-methoxy-N-phenylbenzamide* (**2c**). White solid, yield: 59%. mp: 142–143 °C. ^1^H-NMR (500 MHz, DMSO-*d_6_*) δ: 1.55 (3H, dd, *J* = 6.5 and 20.0 Hz), 3.96 (3H, s), 5.34 (1H, qq, *J* = 7.0 and 48.5 Hz), 7.09 (1H, t, *J* = 8.0 Hz), 7.35 (2H, t, *J* = 8.0 Hz), 7.61 (2H, m), 7.74 (2H, d, *J* = 8.0 Hz), 8.17 (1H, d, *J* = 8.0 Hz), 9.22 (1H, s), 10.12 (1H, s). ESI-HRMS *m/z*: 317.13087 (M+H^+^) (Calcd for C_17_H_18_FN_2_O_3_: 317.13015).

#### 3.1.3. General Procedure for the Synthesis of Compound **3a–g**

Compound **1a/1c/1d** (2.2 mmol) was dissolved in CH_2_Cl_2_ (15 mL), and NaHCO_3_ (2.5 mmol) and MeI/*n*-PrI (22 mmol) were added. The resulting mixture was stirred for 12 h at room temperature. The mixture was filtered, and the filtrate was concentrated *in vacuo* to give a residue which was purified over silica gel (petroleum/ethyl acetate = 2/1) to give the target compounds **3a**–**g**.

*4-Methoxy-3-methylamino-N-phenylbenzamide* (**3a**). White solid, yield: 33%. mp: 170–172 °C. ^1^H-NMR (400 MHz, DMSO-*d_6_*) δ: 2.77 (d, 3H, *J* = 5.2 Hz), 3.84 (s, 3H), 5.19 (q, 1H, *J* = 5.2 Hz), 6.88 (d, 1H, *J* = 8.0 Hz), 7.05 (m, 2H), 7.23 (d, 1H, *J* = 8.0 Hz), 7.32 (t, 2H, *J* = 8.0 Hz), 7.74 (d, 2H, *J* = 8.0 Hz), 9.93 (s, 1H). ^13^C-NMR (100 MHz, CDCl_3_) δ: 30.2, 55.6, 107.8, 108.2, 115.0, 119.9, 124.1, 128.1, 129.0, 138.3, 139.5, 149.6, 166.1. ESI-HRMS *m/z*: 257.12934 (M+H^+^) (Calcd for C_1__5_H_1__7_N_2_O_2_: 257.12900).

*N-(4-Chlorophenyl)-3-dimethylamino-4-methoxybenzamide* (**3b**). White solid, yield: 40%. mp: 160–162 °C. ^1^H-NMR (400 MHz, DMSO-*d_6_*) δ: 2.77 (6H, s), 3.86 (3H, s), 7.03 (1H, d, *J* = 8.0 Hz), 7.37 (2H, d, *J* = 8.0 Hz), 7.43 (1H, s), 7.60 (1H, d, *J* = 8.0 Hz), 7.79 (2H, d, *J* = 8.0 Hz), 10.12 (1H, s). ^13^C-NMR (100 MHz, DMSO-*d_6_*) δ: 23.8, 56.2, 111.4, 117.7, 122.2, 127.2, 127.4, 128.8, 138.9, 142.3, 155.1, 157.3, 165.9. ESI-HRMS *m/z*: 305.10524 (M+H^+^) (Calcd for C_1__6_H_1__8_ClN_2_O_2_: 305.10568).

*4-Methoxy-3-methylamino-N-(3,4,5-trimethoxyphenyl)benzamide* (**3c**). White solid, yield: 30%. mp: 147–148 °C. ^1^H-NMR (400 MHz, DMSO-*d_6_*) δ: 2.77 (3H, s), 3.62 (3H, s), 3.75 (6H, s), 3.84 (3H, s), 5.21 (1H, br), 6.89 (1H, d, *J* = 8.0 Hz), 7.01 (1H, s), 7.24 (3H, m), 9.84 (1H, s). ^13^C-NMR (100 MHz, CDCl_3_) δ: 30.2, 55.6, 56.1, 60.9, 65.8, 97.6, 107.8, 108.6, 114.9, 127.9, 134.4, 134.5, 139.5, 149.6, 153.3, 166.1. ESI-HRMS *m/z*: 347.16079 (M+H^+^) (Calcd for C_1__8_H_23_N_2_O_5_: 347.16070).

*3-Dimethylamino-4-methoxy-N-(3,4,5-trimethoxyphenyl)benzamide* (**3d**). White solid, yield: 21%. mp: 120–122 °C. ^1^H-NMR (400 MHz, DMSO-*d_6_*) δ: 2.76 (6H, s), 3.62 (3H, s), 3.75 (6H, s), 3.87 (3H, s), 7.08 (1H, d, *J* = 8.0 Hz), 7.20 (2H, s), 7.48 (1H, s), 7.82 (1H, d, *J* = 8.0 Hz), 9.98 (1H, s). ^13^C-NMR (100 MHz, CDCl_3_) δ: 30.2, 55.6, 56.1, 56.1, 60.9, 97.9, 107.8, 108.4, 110.2, 115.2, 116.4, 124.8, 127.2, 127.8, 134.2, 153.2, 166.3. ESI-HRMS *m/z*: 361.17621 (M+H^+^) (Calcd for C_1__9_H_25_N_2_O_5_: 361.17635).

*4-Methoxy-N-phenyl-3-(propylamino)benzamide* (**3e**). White solid, yield: 46%. mp: 121–123 °C. ^1^H-NMR (400 MHz, DMSO-*d_6_*) δ: 0.94 (3H, t, *J* = 7.2 Hz), 1.62 (2H, m), 3.09 (2H, m), 3.84 (3H, s), 4.93 (1H, t, *J* = 5.6 Hz), 6.88 (1H, d, *J* = 8.4 Hz), 7.05 (2H, m), 7.23 (1H, d, *J* = 8.4 Hz), 7.31 (2H, t, *J* = 8.0 Hz), 7.73 (2H, d, *J* = 8.0 Hz), 9.93 (1H, s). ^13^C-NMR (100 MHz, DMSO-*d_6_*) δ: 11.6, 21.7, 40.1, 55.6, 107.8, 108.6, 115.6, 120.3, 123.2, 127.4, 128.5, 137.8, 139.4, 148.9, 165.7. ESI-HRMS *m/z*: 285.15975 (M+H^+^) (Calcd for C_17_H_21_N_2_O_2_: 285.16006).

*N-(4-Chlorophenyl)-4-methoxy-3-propylaminobenzamide* (**3f**). White solid, yield: 37%. mp: 168–171 °C. ^1^H-NMR (500 MHz, DMSO-*d_6_*) δ: 0.93 (3H, t, *J* = 7.0 Hz), 1.59 (2H, m), 3.09 (2H, m), 3.84 (3H, s), 4.93 (1H, t, *J* = 5.0 Hz), 6.89 (1H, d, *J* = 8.0 Hz), 7.05 (1H, s), 7.24 (1H, dd, *J* = 1.5 and 8.0 Hz), 7.36 (2H, d, *J* = 8.0 Hz), 7.79 (2H, d, *J* = 8.0 Hz), 10.05 (1H, s). ^13^C-NMR (100 MHz, DMSO-*d_6_*) δ: 11.6, 21.7, 39.9, 55.6, 107.8, 108.7, 115.7, 121.8, 126.8, 127.2, 128.4, 137.9, 138.5, 149.0, 165.9. ESI-HRMS *m/z*: 319.12139 (M+H^+^) (Calcd for C_1__7_H_20_ClN_2_O_2_: 319.12133).

*4-Methoxy-3-(propylamino)-N-(3,4,5-trimethoxyphenyl)benzamide* (**3g**). White solid, yield: 41%. mp: 164–167 °C. ^1^H-NMR (500 MHz, DMSO-*d_6_*) δ: 0.92 (3H, t, *J* = 7.0 Hz), 1.60 (2H, m), 3.09 (2H, m), 3.62 (3H, s), 3.75 (6H, s), 3.84 (3H, s), 4.93 (1H, br), 6.89 (1H, d, *J* = 8.0 Hz), 7.06 (1H, s), 7.23 (3H, m), 9.84 (1H, s). ^13^C-NMR (100 MHz, CDCl_3_) δ: 11.5, 22.5, 45.2, 55.5, 56.0, 60.9, 97.6, 108.2, 108.3, 114.6, 127.8, 134.5, 134.5, 138.7, 149.4, 153.3, 166.1. ESI-HRMS *m/z*: 375.19227 (M+H^+^) (Calcd for C_20_H_27_N_2_O_5_: 375.19200).

*3-Acetoxy-4-methoxybenzoic acid* (**4c**). To a solution of 3-hydroxy-4-methoxybenzoic acid (5.0 g, 30 mmol) and Et_3_N (4.5 g, 45 mmol) in CH_2_Cl_2_ (100 mL) was added acetyl chloride (3.5 g, 45 mmol) at 0 °C, and the resulting mixture was warmed to room temperature and then stirred for another 2 h. The reaction mixture was then washed with water (100 mL), and brine (100 mL) successively. The organic layer was dried over anhydrous MgSO_4_, filtered and concentrated, and the crude residue was purified over silica gel (petroleum/ethyl acetate = 4/1) to give compounds **4c** (3.8 g, yield: 60%), white solid, mp: 230–231 °C. ^1^H-NMR (400 MHz, DMSO-*d_6_*) δ: 2.23 (3H, s), 3.85 (3H, s), 7.35 (1H, d, *J* = 8.0 Hz), 7.83 (1H, s), 7.90 (1H, d, *J* = 8.0 Hz), 12.21 (1H, s). ESI-HRMS *m/z*: 209.04513 (M−H^−^) (Calcd for C_1__0_H_9_O_5_: 209.04500).

*N-(4-Chlorophenyl)-N-methyl-4-methoxy-3-propionyloxybenzamide* (**5d**). A mixture of compound **4b** (0.5 g, 2.23 mmol) and SOCl_2_ (15 mL, 206 mmol) was heated under reflux for 1 h. The excess SOCl_2_ was evaporated and the corresponding chloride product was obtained as yellow oil, which was dissolved in CH_2_Cl_2_ (5 mL) and used without further purification. *N*-methyl-4-chlorobenzenamine (0.38 g, 2.68 mmol) was dissolved in CH_2_Cl_2_ (20 mL), and Et_3_N (0.34 g, 3.34 mmol) was added, and the mixture was cooled to 0 °C (ice bath). A solution of the acid chloride product in CH_2_Cl_2_ (5 mL) was added to the solution dropwise. The resulting mixture was warmed to room temperature and then stirred for another 4 h, after which water (20 mL) was added, and the organic layer was separated, washed with brine (30 mL), and dried over anhydrous MgSO_4_. It was then filtered and concentrated to give a residue, which was purified over silica gel (petroleum/ethyl acetate = 1/1) to yield compound **5d** as a white solid (0.5 g, yield: 64%). mp: 139–141 °C. ^1^H-NMR (400 MHz, CDCl_3_) δ: 1.23 (3H, t, *J* = 7.6 Hz), 2.55 (2H, q, *J* = 7.6 Hz), 3.44 (3H, s), 3.77 (3H, s), 6.72 (1H, d, *J* = 8.4 Hz), 6.98 (1H, d, *J* = 8.4 Hz), 7.06 (1H, s), 7.11 (1H, d, *J* = 8.8 Hz), 7.22 (2H, d, *J* = 8.8 Hz). ESI-HRMS *m/z*: 348.10021 (M+H^+^) (Calcd for C_18_H_19_ClNO_4_: 348.10026).

### 3.2. Biological Activity Test Procedures

Vero cells were purchased from the American Type Culture Collection and cultured in Minimum Essential Medium (MEM) supplemented with 10% Fetal Bovine Serum (FBS) and antibiotics (100 U/mL penicillin G, 100 μg/mL streptomycin). Enterovirus 71 (EV 71) strain SZ-98 was kindly provided by Dr. Qi Jin, Institute of Pathogen Biology, Chinese Academy of Medical Science and Peking Union Medical School. Enterovirus 71 (EV 71) strain BrCr (VR-1775) was purchased from the American Type Culture Collection. Enterovirus 71 (EV 71) strain JS-52-3 was kindly provided by Dr. Xiang-zhong Ye, Beijing Wantai Biological Pharmacy Enterprise Co., Ltd. All Enterovirus 71 strains were propagated in Vero cells.

All compounds were dissolved in DMSO at 20 mg/mL as a stock solution and further diluted in culture medium prior to use. The initial concentration in all cytotoxicity assay was 200 µg/mL, and inhibition assay for anti-EV71 experiments was from maximum non-toxic concentration, and proceeded serially 3-fold dilution with culture medium. All cytotoxicity and activity assay for each compound was repeated for three times, and the data was indicated as average ± SD.

#### 3.2.1. Cytotoxicity Determination

The cytotoxicity of compounds in the presence of Vero cells was monitored by cytopathic effect (CPE). Vero cells (2.5 × 10^4^/well) were plated into a 96-well plate. A total of 24 h later, the monolayer cells were incubated in the presence of various concentrations of test compounds. After 48 h of culture at 37 °C and 5% CO_2_ in a carbon-dioxide incubator, the cells were monitored by CPE. The median toxic concentration (TC_50_) was calculated by Reed & Muench analyses.

#### 3.2.2. Anti EV 71 Activity Assay

Confluent Vero cells grown in 96-well microplates were infected respectively with 100 median tissue culture infective dose (TCID_50_) EV 71. After 1 h adsorption at 37 °C, the monolayers were washed by PBS and incubated at 37 °C in the maintenance medium with or without different concentrations of test compounds. Viral cytopathic effect (CPE) was observed when the viral control group reached 4 and the antiviral activity of tested compounds was determined by the Reed & Muench analyses.

## 4. Conclusions

In summary, a series of *N*-phenylbenzamide derivatives were synthesized and their anti-EV 71 activities were evaluated *in vitro*. All of the synthesized compounds were less toxic to Vero cells in comparison to pirodavir, and several compounds were comparable or superior to pirodavir in terms of SI values, especially compound **1e**. This compound has a molecular weight and an ALogP of 321.169 g/mol and 2.6 (data obtained from Discovery Studio 3.0), respectively, and shows potential for optimization to improve its activity against EV 71 without sacrificing its druggability. Consequently, compound **1e** is a promising lead compound for the development of a new anti-EV 71 agent, and further lead optimization based on compound **1e** will be performed.
